# Eye-Tracking Reveals that the Strength of the Vertical-Horizontal Illusion Increases as the Retinal Image Becomes More Stable with Fixation

**DOI:** 10.3389/fnhum.2017.00143

**Published:** 2017-03-24

**Authors:** Philippe A. Chouinard, Hayden J. Peel, Oriane Landry

**Affiliations:** Department of Psychology and Counselling, School of Psychology and Public Health, La Trobe UniversityMelbourne, VIC, Australia

**Keywords:** vertical-horizontal illusion, size perception, eye tracking, spatial attention, framing theory, misapplied constancy scaling theory

## Abstract

The closer a line extends toward a surrounding frame, the longer it appears. This is known as a framing effect. Over 70 years ago, Teodor Künnapas demonstrated that the shape of the visual field itself can act as a frame to influence the perceived length of lines in the vertical-horizontal illusion. This illusion is typically created by having a vertical line rise from the center of a horizontal line of the same length creating an inverted T figure. We aimed to determine if the degree to which one fixates on a spatial location where the two lines bisect could influence the strength of the illusion, assuming that the framing effect would be stronger when the retinal image is more stable. We performed two experiments: the visual-field and vertical-horizontal illusion experiments. The visual-field experiment demonstrated that the participants could discriminate a target more easily when it was presented along the horizontal vs. vertical meridian, confirming a framing influence on visual perception. The vertical-horizontal illusion experiment determined the effects of orientation, size and eye gaze on the strength of the illusion. As predicted, the illusion was strongest when the stimulus was presented in either its standard inverted T orientation or when it was rotated 180° compared to other orientations, and in conditions in which the retinal image was more stable, as indexed by eye tracking. Taken together, we conclude that the results provide support for Teodor Künnapas’ explanation of the vertical-horizontal illusion.

## Introduction

**Figure 1:**
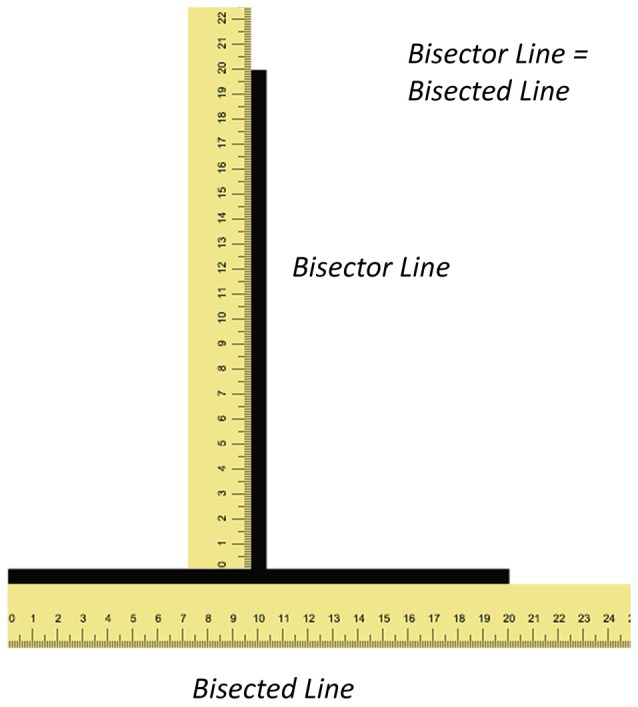
**Illustrations depicting the illusion and shape of the visual field.** The figure illustrates the illusion with rulers (not to scale) along both lines. Both the vertical (bisector) and horizontal (bisected) lines have the same length yet the former appears longer than the latter.

The vertical-horizontal illusion typically consists of a vertical line rising from the center of a horizontal line creating an inverted T figure (Figure 1). Although both lines are of equal length, the vertical line appears longer than the horizontal one. The most prominent theory to explain this illusion was proposed by Künnapas (1955). This theory, known as the framing theory, is based on the principle that the shape of the visual field is more elongated in the horizontal plane as a result of the combined visual fields from each eye merging together. This shape of the visual field under binocular vision creates a framing effect whereby a vertical line appears longer than a horizontal line of the same length. This is because the former is closer to the boundaries of the visual field than the latter.

The framing theory is supported under a number of experimental settings. The illusion is more powerful when it is presented in its most common orientation (Figure 1) than when it is rotated 90° (Finger and Spelt, 1947; Harris et al., 1974; Thompson and Schiffman, 1974; Charras and Lupiáñez, 2010) or when the former configuration is viewed with the head rotated upright compared to when the head is rotated 90° (Künnapas, 1955; Avery and Day, 1969). Likewise, the illusion is stronger when an elongated artificial frame is presented over the figure in the horizontal compared to vertical orientation (Künnapas, 1957; Prinzmetal and Gettleman, 1993). Künnapas (1959) also manipulated the shape of the participant’s field of view with special goggles and revealed that the illusion was strongest when the field of view was elongated in the horizontal compared to the vertical plane.

The illusion is also stronger under binocular than monocular viewing conditions (Prinzmetal and Gettleman, 1993) and is believed to rely on cortical mechanisms, where information from both eyes first begin to merge (Hubel and Wiesel, 1962; Qian, 1997; Scholl et al., 2013). In support of the importance of cortical mechanisms, Harris et al. (1974) have shown that the illusion is as strong during dichoptic relative to binocular viewing conditions, which would not be possible if the illusion was largely mediated by retinal processing. In addition, the illusion can be induced by haptic exploration without visual feedback (Avery and Day, 1969; Deregowski and Ellis, 1972) and can also be enhanced with perspective cues (Girgus and Coren, 1975; Wolfe et al., 2005).

Despite all this, there are certain aspects of the illusion that the framing theory cannot explain. For example, the theory cannot explain why bisecting the lines any differently, such as somewhere other than the center, causes a less powerful illusion (Harris et al., 1974) nor can it explain why the illusion is still present when people explore the stimuli haptically under conditions without visual feedback (Avery and Day, 1969; Deregowski and Ellis, 1972). Nevertheless, the framing theory remains the most parsimonious and best known account of the vertical-horizontal illusion. For alternative accounts, see Girgus and Coren (1975), Mamassian and de Montalembert (2010) and Mikellidou and Thompson (2013).

Position constancy is the perceptual experience of seeing a visual scene remaining stationary despite eye movements. It depends on processes that continuously monitor and send oculomotor signals to the perceptual regions of the cortex in order to update a perceptual experience that is stable (Feldman, 2016). We reason that a framing effect would not be possible without the creation of a stable perceptual outside world, which includes perceiving stable boundaries of the visual field. We also reason that if the frame created from the perceptual shape of the visual field is important for the vertical-horizontal illusion then the illusion would be stronger under conditions where the computational demands for achieving position constancy is reduced, such as when the retinal image is more stabilized from maintaining fixation on a particular spatial location. In cases of gross saccadic eye movements, such as when participants are asked to gaze freely where they want to, the location of the boundaries of the visual field are constantly in flux both in retinal and perceptual space, reducing the strength of any framing effect.

In the present study, we repeated some of the classical experimental conditions that were instrumental for developing the framing theory but also had participants either fixate at the intersection of the stimulus or gaze wherever they liked on the computer screen while we recorded their eye movements with an eye tracker. As far as we know, nobody has done eye tracking before in previous investigations of the vertical-horizontal illusion. In order to explore the effects of retinal image stability on this phenomenon, we performed two experiments: the visual-field and vertical-horizontal illusion experiments.

The visual-field experiment aimed to confirm the existence of a framing effect. Should a framing effect exist, one might expect a target to be detected more easily at a particular distance away from fixation, or eccentricity, along the horizontal compared to the vertical meridian. Thus, in this experiment, we presented a target at one of two different eccentricities, one inside and the other outside of the fovea, at a location above, below, to the left or right of fixation. Given the elongated shape of the visual field in the horizontal direction, we hypothesized that people would be better at detecting the target when presented along the horizontal than the vertical meridian at the more peripheral eccentricity. In addition, if one considers that the extent of the upper is smaller than the lower visual field (Niederhauser and Mojon, 2002) then one could also predict that people might be better at detecting the target when it is presented below compared to above fixation at the more peripheral eccentricity. We also predicted that there would be no differences in eye gaze across conditions given that participants were instructed to always maintain fixation.

The aims of the vertical-horizontal illusion experiment were twofold. First, we sought to determine the effects of orientation, size and eye gaze on the strength of the illusion. Based on the framing theory, we hypothesized that the illusion would be stronger when the stimulus is presented in either its standard inverted T orientation or when it is rotated 180° compared to the other orientations. Regarding size, we predicted that the illusion would be stronger when the stimulus was smaller based on previous work investigating the effects of size on illusion strength (Thompson and Schiffman, 1974). Regarding eye gaze, we predicted that the illusion would be stronger when participants fixated at the intersection of the two lines compared to when they are asked to free gaze. The latter would conceivably result in greater eye movements, greater demands on position constancy mechanisms and weaker framing effects. Second, we sought to characterize how spatial attention may differ between the different conditions and determine if these differences may explain illusion susceptibility. We did not have any specific predictions with regards to this second aim.

## Materials and Methods

### Participants

Twenty participants (6 males, mean age = 23.2 years, age range = 18–38 years) participated in the visual field and vertical-horizontal illusion experiments. Eighteen participants reported to be right-handed while two participants reported to be left handed. All participants reported to have normal or corrected-to-normal vision. None of the participants reported a history of neurological impairment. Out of the 20 participants, eye-tracking was recorded in 10 (4 males, mean age = 22.9 years, age range = 18–38 years). All participants reported to be right handed. In accordance with the Declaration of Helsinki, the procedures were approved by the La Trobe University Human Ethics Committee. All participants provided informed written consent prior to participation and received either course credit or monetary compensation for their time.

### Apparatus

We presented stimuli for all experiments on a Tobii 23-inch liquid-crystal display monitor at a resolution of 1600 × 900 pixels subtending 46.0° × 26.3° of visual angle (Tobii AB, Stockholm, Sweden). The screen refresh rate was 60 Hz. Eye tracking was carried out using the Tobii Pro TX300 eye-tracking system (Tobii AB, Stockholm, Sweden) in 10 of the 20 participants. All 20 participants, regardless as to whether or not they had eye-tracking, kept their head in a chin rest such that the viewing distance was 60 cm. Manual responses were recorded using the numerical keypad on a standard computer keyboard. Both the presentation of the stimuli and the eye tracking were controlled by a Dell Precision M6800 mobile workstation (Dell, Round Rock, TX, USA) using program scripts written in E-Prime 2.0 software and extension suites for Tobii (Psychology Software Tools, Pittsburgh, PA, USA).

### Procedure for the Visual-Field Experiment

**Figure 2:**
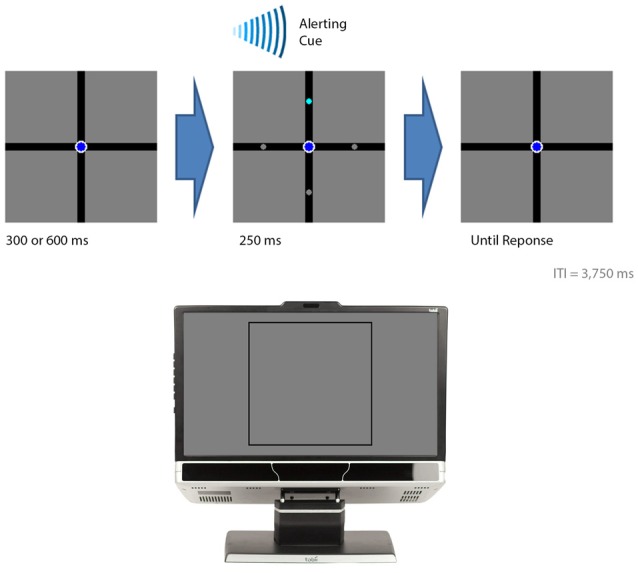
**The events in the visual-field experiment.** The figure illustrates the sequential events for a trial. The bottom section of the figure illustrates where on the computer monitor the displays presented in the top sections of the figure appeared. A trial began with either a 300 or 600 ms delay followed by the presentation of the target (cyan circle) with three distractor stimuli (gray circles). An auditory beep alerted the participant to the presence of the target. The target was presented for 250 ms. The participant indicated the location of the target by button pressing. In this example, the target appeared in the upper visual field and the participant was expected to press “8” on a numerical keypad.

The background display consisted of a black (luminance: 0.1 cd/m^2^) cross over a gray background (luminance: 12.3 cd/m^2^; Figure 2). The cross was 0.6° thick and subtended 22.6° of visual angle in both the horizontal and vertical directions. On a given trial, a circle was presented on each arm: three gray (luminance: 12.3 cd/m^2^) and one cyan (luminance: 44.0 cd/m^2^). The participant’s task was to indicate which of the four arms briefly displayed the cyan circle. The circles were 0.2° in diameter. The participant was also instructed to maintain fixation on a navy blue (luminance: 5.1 cd/m^2^) fixation circle (0.4° in diameter) positioned over the intersection of the cross during the entire experiment.

Figure 2 provides the temporal sequence of events for each trial. Each trial began with a delay of either 300 or 600 ms. This was followed by the brief presentation of the circles for 250 ms while an auditory cue, consisting of a beep, alerted their presence. The circles appeared either 1.9° (the foveal condition) or 7.2° (the peripheral condition) from fixation. The cyan circle appeared 10 times in each position, giving rise to an overall total of 80 trials for the entire experiment. E-prime randomly determined the order of where the cyan circle was presented for each participant. The participant pressed “4” when they perceived the cyan circle in the left arm, “8” for the top arm, “5” for the bottom arm, and “6” for the right arm. The participant had 2500 ms to make a response otherwise the trial was coded as an error. An inter-trial interval of 3750 ms followed after the circles disappeared. A practice block was carried out before the experiment in which the cyan circle was presented once in each position, giving rise to an overall total of 8 practice trials. This served to familiarize the participants with the procedures before the actual data were collected.

### Procedure for the Vertical-Horizontal Illusion Experiment

**Figure 3:**
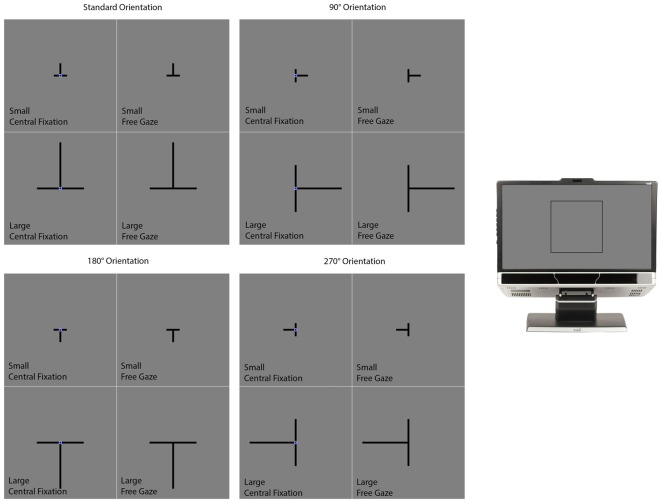
**The conditions in the vertical-horizontal illusion experiment.** The section on the far right of the figure illustrates where on the computer monitor the displays presented in the rest of the figure appeared. The experiment had a 2 (Size) × 2 (Gaze) × 4 (Orientation) design consisting of the 16 conditions illustrated in this figure. On all trials, the two lines were initially presented with the same length. One of the lines was designated as the standard while the other was designated as the comparison stimulus. The standard did not change in length and the participant’s task was to adjust the length of the comparison stimulus to match the standard. This was accomplished by pressing “4” or “6” on the numerical keypad to make the comparison stimulus smaller or larger respectively. Participants informed the experimenter when they felt they had matched the comparison stimulus to the standard stimulus. The experimenter then advanced the program to the next trial. Participants were given as much time as they needed to complete each trial. The participant’s final adjustment was measured in pixels.

The background of the display was gray (luminance: 12.3 cd/m^2^). Each trial began with a delay of 2000 ms followed by the presentation of the illusion in the form of a T presented in different sizes and orientations (Figure 3). The intersection of the T was positioned at the center of the screen. The two lines were 0.3° thick and always had the same length—subtending either 1.9° (the small condition) or 7.2° (the large condition) of visual angle (Figure 3). The T was presented in one of four different orientations (Figure 3). The first consisted of the T inverted, which is how the illusion is most commonly presented. Under this “standard” orientation, the vertical line typically appears longer than the horizontal one when both are physically the same. The other orientations consisted of the T rotated 90°, 180° and 270° from the standard orientation.

The method of adjustment was used to measure the perceived length of the lines in a manner similar to the one used in our previous studies (Chouinard et al., 2013, 2016). This method was favored over other methods (e.g., the method of constant stimuli) so that the participants were free to scan the visual scene without any temporal constraints. One of the lines was designated as the standard feature while the other was designated as the comparison feature. The standard feature did not change in length and the participant’s task was to adjust the comparison feature to match the standard feature. This was accomplished by pressing “4” or “6” on the numerical keypad to make the comparison feature shorter or longer respectively. Changes were made in increments of 0.06° of visual angle. This consisted of adding or removing one pixel from each end of the bisected line and adding or removing two pixels from the far end of the bisector line. The participant carried out the task at their own pace and were given as much time as they needed to complete each trial. At the end of each trial, the participant informed the experimenter when they felt they had finally matched the comparison feature to the standard feature. The participant’s final adjustment was recorded in pixels by the computer program and the experimenter then advanced the program to the next trial.

A navy blue (luminance: 5.1 cd/m^2^) fixation circle (0.5° in diameter) was positioned over the intersection of the lines in some trials (the central fixation condition) but not in others (the free gaze condition; Figure 3). The presence of the fixation circle cued the participant where they should position their gaze. Otherwise, the participant was free to move their gaze wherever they liked on the computer screen. Figure 3 illustrates the different conditions. The conditions differed in size (small or large), orientation (standard, 90°, 180°, or 270°) and gaze (central fixation or free gaze). There were two trials per condition for a total of 32 trials. For a given condition, the two trials differed with respect to which lines were designated as the standard and comparison features. E-Prime randomly generated the order of the different conditions for each participant.

### Eye Tracking

For the participants who had eye tracking recorded, a calibration procedure was performed at the beginning of the test session. During this procedure, the participant was asked to track with their eyes a red circle, subtending 1.7° of visual angle, that moved between nine positions on the computer screen (top left, center and right; middle left, center and right; bottom left, center and right). After this registration, the system recorded the data in X, Y Cartesian coordinates relative to the top left corner of the computer screen in pixels at a sample rate of 300 Hz. The data were converted to degrees of visual angle from the eye tracker’s coordinate space and then smoothed using a 3-unit median filter. After preprocessing the data in this manner, we calculated the following dependent variables: gaze deviation from center, saccade count, trial duration (for the vertical-horizontal illusion experiment only), proportion of gaze along the horizontal meridian, and proportion of gaze along the vertical meridian.

The first of these variables consisted of the amount of eye-gaze deviation from fixation, which we calculated as the average Euclidian distance from the center of the computer screen of every frame collected. For saccade counts, we first identified saccades from the data using an automated procedure described by Engbert and Kliegl (2003) and counted the number of saccades that were flagged for each trial. For flagging instances of saccades, we considered the change in velocity over time and synchrony of both eyes using the following parameters: time span around sample to compute velocity: 20 ms; velocity threshold to detect saccade: 6× the median velocity of all frames collected; minimum duration for a saccade: 12 ms; minimal time separating saccades: 12 ms. Last, we counted the number of frames in which the eyes were positioned within 0.5° of visual angle from the horizontal and vertical meridians and converted these scores to a percentage of frames collected in each trial.

### Statistical Analyses

For the visual-field experiment, we entered the dependent variables in a repeated-measures analysis of variance (ANOVA) with Visual Field (left vs. upper vs. right vs. bottom) and Eccentricity (foveal vs. peripheral) as factors. These analyses were performed in all 20 participants for the accuracy (%) and reaction time (ms) measures and in the 10 participants who underwent eye tracking for the gaze deviation from center (degrees of visual angle), saccade count (n) and the amount of gaze along the horizontal and vertical meridians (% of frames from total frames collected in each trial) measures.

For the vertical-horizontal illusion experiment, we calculated for all 20 participants a normalized index of susceptibility, which allows for more meaningful comparisons of illusion susceptibility across studies. This was calculated in a manner similar to what we and others have done in the past (e.g., Schwarzkopf et al., 2011; Buckingham and Goodale, 2013; Chouinard et al., 2013, 2016); namely: (Final Adjustment for the Bisected Line to Match the Bisector Line − Final Adjustment for the Bisector Line to Match the Bisected Line)/(Final Adjustment for the Bisected Line to Match the Bisector Line + Final Adjustment for the Bisector Line to Match the Bisected Line; Figure 1). A one-sample *t* test against zero and a Cohen’s *d* effect size score were calculated for each condition. Bonferroni corrections were applied to these tests to account for the 16 conditions.

The susceptibility scores were also entered in a repeated-measures ANOVA with Orientation (standard vs. 90° vs. 180° vs. 270°), Gaze (central fixation vs. free gaze) and Size (small vs. large) as factors. The same ANOVA was also applied for each of the eye-tracking dependent variables (i.e., saccade count, trial duration, amount of gaze along the horizontal and vertical meridians) collected in the 10 participants who had eye tracking. In addition, we performed a series of correlations to determine if the susceptibility index for a given condition was associated with any eye-tracking measurements.

For the ANOVA carried out in the two experiments, Greenhouse-Geisser corrections were applied whenever the assumption of sphericity was not met according to a Mauchly’s sphericity test. Simple effects tests and Tukey’s HSD pair-wise comparison tests, which corrected for multiple comparisons, were carried out *post hoc* to delineate the more complicated interactions and effects deemed significant by the ANOVA. All reported *p* values accounted for multiple comparisons, and statistical significance was evaluated in reference to an alpha level of 0.05.

## Results

### Visual-Field Experiment

The statistical results for the visual-field experiment that are presented in the following sections reveal the following picture. The participants seemed to have more difficulty detecting targets when they were presented along the vertical meridian and when they were presented in the periphery. The eye tracking results demonstrated that the participants were generally good at maintaining fixation at the center of the screen yet tended to deviate more along the vertical than the horizontal meridian. Also, more saccades were made when the target appeared in the periphery compared to the fovea.

### Accuracy (% Correct)

**Figure 4:**
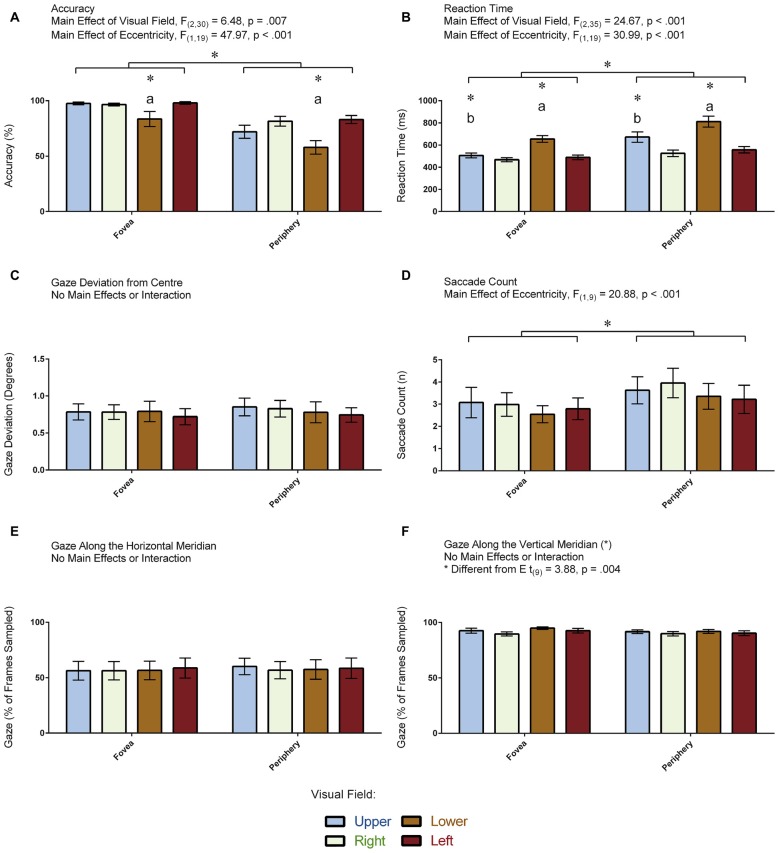
**Results for the visual-field experiment.** The figure depicts the means and standard errors (SEM) for each condition for each of the following dependent variables: **(A)** accuracy (% correct); **(B)** reaction time (ms); **(C)** gaze deviation from center (degrees of visual angle); **(D)** saccade count (*n*); **(E)** gaze along the horizontal meridian (% of frames sampled); and, **(F)** gaze along the vertical meridian (% of frames sampled). All asterisks (*) denote significant differences after corrections were applied for multiple comparisons (*p*_corr_ < 0.05). Labeled asterisks (*) denote differences from: (a) the upper, right and left visual fields for that eccentricity condition; and, (b) the lower, right and left visual fields for that eccentricity condition.

Figure 4A displays the accuracy measurements that were obtained for each condition in the 20 participants. ANOVA revealed a main effect of Visual Field: *F*_(2,31)_ = 6.48, *p* = 0.007 (Greenhouse-Geisser corrected). Pair-wise comparisons revealed that participants were less accurate in indentifying the target in the lower compared to the other visual fields (all *p* < 0.035). No other pair-wise comparisons were significant (all *p* > 0.661). ANOVA also revealed a main effect of Eccentricity: *F*_(1,19)_ = 47.96, *p* < 0.001, denoting more errors when the target appeared in the periphery compared to the fovea. The Visual Field × Eccentricity interaction was not significant: *F*_(3,57)_ = 2.56, *p* = 0.064. An independent samples *t*-test demonstrated greater accuracy in the participants who had eye tracking (*M* = 91.38, *SD* = 3.97) compared to those who did not (*M* = 76.12, *SD* = 8.87; *t*_(18)_ = 4.96, *p* < 0.001). In summary, performance was reduced when the target appeared in the lower visual field and in the periphery.

### Reaction Time (ms)

Figure 4B displays the reaction time measurements that were obtained for each condition in the 20 participants. ANOVA revealed a main effect of Visual Field: *F*_(2,35)_ = 24.67, *p* < 0.001 (Greenhouse-Geisser corrected). Pair-wise comparisons revealed that participants were slowest to respond when the target appeared in the lower compared to the other visual fields (all *p* < 0.001) and slower to respond when the target appeared in the upper compared to the right visual field (*p* = 0.017). No other pair-wise comparisons were significant (all *p* > 0.140). ANOVA also revealed a main effect of Eccentricity: *F*_(1,19)_ = 30.99, *p* < 0.001, denoting slower responses when the target appeared in the periphery relative to the fovea. The Visual Field × Eccentricity interaction was not significant: *F*_(2,35)_ = 3.15, *p* = 0.059 (Greenhouse-Geisser corrected). An independent samples *t*-test demonstrated no differences in reaction time to the illusion between the participants who completed the task with (*M* = 580.34, *SD* = 50.58) and without (*M* = 590.63, *SD* = 125.32) eye tracking (*t*_(18)_ = 0.24, *p* = 0.812). In summary, participants were slower to identify the target when it appeared along the vertical meridian as well as when it appeared in the periphery.

### Checking for Speed-Accuracy Trade-Offs

The accuracy and reaction time for each of the 20 participants were averaged across all conditions and correlated with each other to determine if there were any speed-accuracy trade-off effects. This analysis did not yield a significant correlation (*r*_(18)_ = −0.24, *p* = 0.310). Thus, speed-accuracy trade-off effects did not seem to be present in this experiment. Namely, accuracy did not decrease in participants who responded more quickly.

### Gaze Deviation from Center (Degrees of Visual Angle)

Figure 4C displays the amount of gaze deviation from the center of the screen for each condition in the 10 participants who had eye tracking. ANOVA did not reveal a main effect of Visual Field (*F*_(2,14)_ = 0.16, *p* = 0.804, Greenhouse-Geisser corrected) nor did it reveal a main effect of Eccentricity (*F*_(1,9)_ = 1.22, *p* = 0.298). Likewise, the Visual Field × Eccentricity interaction was not significant: *F*_(2,16)_ = 0.35, *p* = 0.676 (Greenhouse-Geisser corrected). In summary, gaze deviation from the center of the screen was the same across all conditions.

### Saccade Count (*n*)

Figure 4D displays the number of saccades made per trial in the 10 participants for whom eye tracking was recorded. ANOVA revealed a main effect of Eccentricity: *F*_(1,9)_ = 20.88, *p* < 0.001, denoting more saccades when the target was in the periphery compared to the fovea, but it did not reveal a main effect of Visual Field: *F*_(3,27)_ = 1.86, *p* = 0.159. In addition, the Visual Field × Eccentricity interaction was not significant: *F*_(3,27)_ = 0.33, *p* = 0.803. In summary, participants made more saccades when the target appeared in the periphery.

### Gaze Along the Horizontal Meridian (% of Frames Sampled)

Figure 4E displays the proportion of frames where the gaze was directed along the horizontal meridian for each condition in the 10 participants for whom eye tracking was recorded. ANOVA did not reveal a main effect of Visual Field (*F*_(3,27)_ = 0.34, *p* = 0.793) nor did it reveal a main effect of Eccentricity (*F*_(1,9)_ = 3.23, *p* = 0.104). Likewise, the Visual Field × Eccentricity interaction was not significant: *F*_(3,27)_ = 0.44, *p* = 0.723. In summary, participants directed their gaze along the horizontal meridian to the same degree across all conditions—regardless whether the target appeared at a location in the fovea or periphery that was above, below, to the left or to the right of fixation.

### Gaze Along the Vertical Meridian (% of Frames Sampled)

Figure 4F displays the proportion of frames where the gaze was directed along the vertical meridian for each condition in the 10 participants for whom eye tracking was recorded. ANOVA did not reveal a main effect of Visual Field (*F*_(3,27)_ = 2.01, *p* = 0.137) nor did it reveal a main effect of Eccentricity (*F*_(1,9)_ = 3.49, *p* = 0.095). Likewise, the Visual Field × Eccentricity interaction was not significant: *F*_(3,27)_ = 0.57, *p* = 0.639. We also carried out a paired *t*-test to assess differences between the proportion of frames where the gaze was directed along the vertical vs. horizontal meridians, which revealed that participants spent more time directing their gaze along the vertical meridian (*t*_(9)_ = 3.88, *p* = 0.004). Note that the means between the meridians do not add up to zero (Figures 4E,F). This is because the participants spent some of their time directing their gaze at the center of the screen where both meridians intersect. In summary, participants directed their gaze along the vertical meridian to the same degree across all conditions—regardless whether the target appeared at a location in the fovea or periphery that was above, below, to the left or to the right of fixation.

### Vertical-Horizontal Illusion Experiment

The statistical results for the vertical-horizontal line experiment that will be presented in the following sections reveal the following picture. The illusion was strongest when the participants were asked to fixate at the center of the screen and when the stimulus was presented in the standard and 180° orientations relative to the 90° and 270° orientations. The illusion was also strongest when it was small. The eye tracking data confirmed that the participants made fewer saccades and did not deviate as much from the center of the screen when they were asked to fixate there. The eye tracking data also revealed that this retinal image stability tended to decrease as the stimulus got larger and was presented in the standard orientation relative to the other orientations. Last, we reveal how the illusion is stronger in conditions when participants made fewer saccades and spent more time gazing along the vertical meridian.

### Illusion Susceptibility

**Figure 5:**
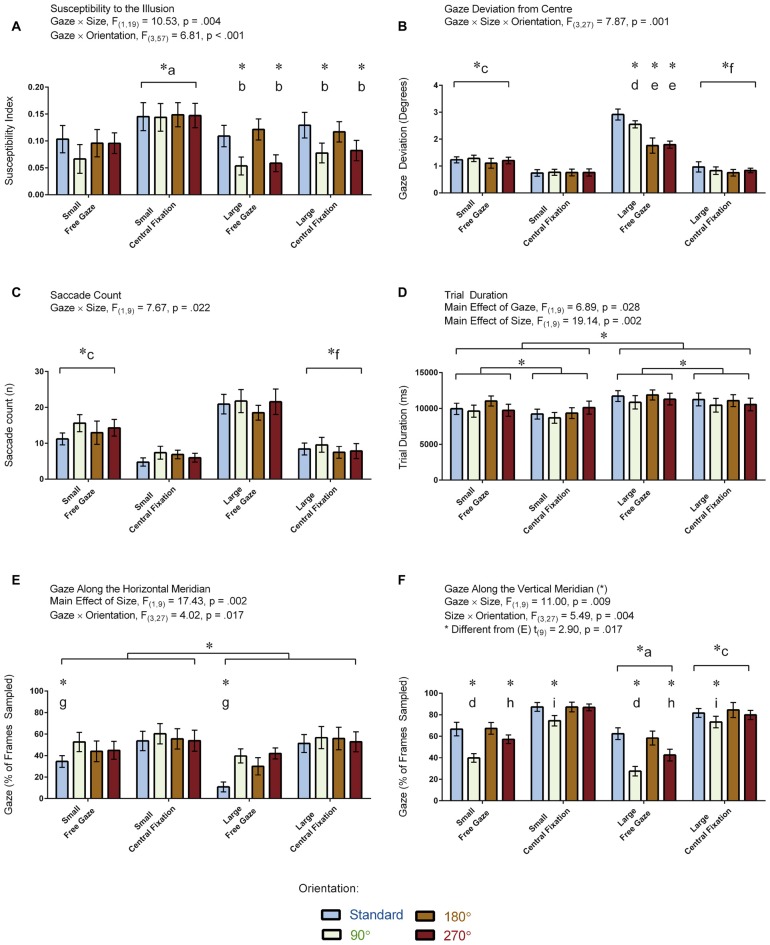
**Results for the vertical-horizontal illusion experiment.** The figure depicts the means and standard errors (SEM) for each condition for each of the following dependent variables: **(A)** illusion susceptibility index; **(B)** gaze deviation from center (degrees of visual angle); **(C)** saccade count (*n*); **(D)** trial duration (ms); **(E)** gaze along the horizontal meridian (% of frames sampled); and, **(F)** gaze along the vertical meridian (% of frames sampled). All asterisks (*) denote significant differences after corrections were applied for multiple comparisons (*p*_corr_ < 0.05). Labeled asterisks (*) denote differences from: (a) the small free gaze and large central fixation conditions; (b) the standard and 180° orientations for that size × gaze condition; (c) the small central fixation and large free gaze conditions; (d) the standard, 180° and 270° orientations for that size × gaze condition; (e) the standard and 90° orientations for that size × gaze condition; (f) the large free gaze conditions; (g) the 90°, 180° and 270° orientations for that size × gaze condition; (h) the standard, 90° and 180° orientations for that size × gaze condition; and, (i) the 180° orientation for that size × gaze condition.

Figure 5A displays illusion susceptibility for each condition in the 20 participants while Table 1 provides descriptive statistics and effect sizes for each condition. One sample *t*-tests reveal that the participants were susceptible to the illusion across all conditions except for when the stimulus was presented in the 90° orientation during free gaze for both the small (*p* = 0.362) and large (*p* = 0.075) condition. ANOVA revealed Gaze × Size (*F*_(1,19)_ = 10.53, *p* = 0.004) and Size × Orientation (*F*_(3,57)_ = 6.81, *p* < 0.001) interactions. All other interactions were not significant (*p* > 0.248) but all main effects were significant (all *p* < 0.006). Our *post hoc* analyses focused on the delineation of the significant two-way interactions. These revealed that the Gaze × Size interaction was driven by increased illusion susceptibility in the central fixation compared to the free gaze condition in the small (*p* < 0.001) but not in the large (*p* = 0.105) condition. This interaction was also driven by increased illusion susceptibility in the small compared to the large condition in the central fixation (*p* < 0.001) but not in the free gaze (*p* = 0.586) condition. As for the Size × Orientation interaction, this was driven by increased susceptibility when the stimulus was oriented in the standard and 180° configurations compared to the 90° and 270° configurations but only when the stimulus was large (all *p* < 0.003). No effects of orientation were found when the stimulus was small (*p* = 0.472). An independent samples *t*-test demonstrated similar susceptibility to the illusion between the participants with (*M* = 0.09, *SD* = 0.09) and without (*M* = 0.12, *SD* = 0.06) eye tracking recorded (*t*_(18)_ = 0.96, *p* = 0.352). In summary, susceptibility was strongest when the participants were asked to fixate at the center of the screen when the stimulus was small and when the large stimulus was oriented in the standard and 180° configurations.

**Table 1:** **Descriptive statistics and effect sizes for the illusion susceptibility index for each condition in the vertical-horizontal illusion**.

Condition			*M*	*SD*	*t*_(19)_	95% *CI*	Cohen’s *d*
Free gaze	Small	Standard	0.10	0.11	4.07*	0.05–0.16	0.91
		90°	0.07	0.12	2.48	0.01–0.12	0.56
		180°	0.10	0.11	3.76*	0.04–0.15	0.84
		270°	0.10	0.09	4.97*	0.06–0.14	1.11
	Large	Standard	0.11	0.09	5.47*	0.07–0.15	1.22
		90°	0.05	0.07	3.20	0.02–0.09	0.72
		180°	0.12	0.09	6.18*	0.08–0.16	1.38
		270°	0.06	0.07	3.71*	0.03–0.09	0.83
Central fixation	Small	Standard	0.15	0.12	5.54*	0.09–0.20	1.24
		90°	0.14	0.11	5.60*	0.09–0.20	1.25
		180°	0.15	0.10	6.63*	0.10–0.20	1.48
		270°	0.15	0.10	6.46*	0.10–0.19	1.44
	Large	Standard	0.13	0.11	5.44*	0.08–0.18	1.22
		90°	0.08	0.08	4.24*	0.04–0.12	0.95
		180°	0.12	0.08	6.19*	0.08–0.16	1.38
		270°	0.08	0.09	4.32*	0.04–0.12	0.97

### Gaze Deviation from Center (Degrees of Visual Angle)

Figure 5B displays the amount of gaze deviation from the center of the screen for each condition in the 10 participants for whom eye tracking was recorded. ANOVA revealed a Gaze × Size × Orientation interaction: *F*_(3,27)_ = 7.87, *p* = 0.001. All two-way interactions and main effects were also significant (*p* < 0.018). We focused our *post hoc* analyses on the delineation of the three-way interaction. Differences between orientation conditions were found only when the stimulus was large and when the participants were free gazing. Specifically, gaze deviation from the center was greater when the stimulus was in its standard orientation relative to the other orientations in the large free gazing condition (all *p* < 0.045). Gaze deviation from the center was also greater for the 90° orientation relative to the 180° and 270° orientations in the large free gazing condition (both *p* < 0.001). In addition, gaze deviation from the center was greater for the large relative to the small condition for each orientation during the free gaze condition (all *p* < 0.001) whereas this was only the case for the standard orientation (*p* = 0.045) but not the other orientations (all *p* > 0.508) in the central fixation condition. We also found that gaze deviation from the center was greater in the free gaze relative to the central fixation condition for each orientation regardless whether the stimulus was small or large (all *p* < 0.023). In summary, gaze deviation from the center was greatest during free gazing and when the stimulus was large, and presenting the stimulus in its standard orientation caused even more gaze deviation from the center during the free gazing of the large stimulus.

### Saccade Count (*n*)

Figure 5C displays the number of saccades made per trial in the 10 participants who had eye tracking. ANOVA revealed a Gaze × Size interaction: *F*_(1,9)_ = 7.67, *p* = 0.022. No other interactions were significant (*p* > 0.076). There were main effects of Gaze and Size (*p* < 0.001) but not Orientation (*p* = 0.100). Our *post hoc* analyses focused on the delineation of the significant interaction. These analyses revealed that the Gaze × Size interaction was driven by more saccades in the large compared to the small condition for the free gaze (*p* < 0.001) but not central fixation (*p* = 0.145) condition. The *post hoc* analyses also revealed that while more saccades were made in the free gaze compared to the central fixation condition for both the small (*p* = 0.002) and large (*p* < 0.001) conditions, these differences were more pronounced in the latter. In summary, the participants made more saccades during free gazing and when the large stimulus was presented.

### Trial Duration (ms)

Figure 5D displays trial durations for each condition in the 10 participants who had eye tracking. Trial durations were not recorded in the other participants because this measure was calculated from the eye tracking data and we had no other means to extrapolate this information from the other recordings. ANOVA revealed a main effect of Gaze: *F*_(1,19)_ = 6.89, *p* = 0.028, denoting shorter trial durations in the central fixation relative to the free gaze condition. ANOVA revealed a main effect of Size: *F*_(1,19)_ = 19.14, *p* = 0.002, denoting shorter trial durations in the small relative to the large condition. The main effect of Orientation was not significant (*F*_(2,17)_ = 3.00, *p* = 0.082, Greenhouse-Geisser corrected). Likewise, all interactions were not significant (all *p* > 0.128). In summary, trial duration was shortest when participants were asked to maintain fixation and when the stimulus was small.

### Gaze Along the Horizontal Meridian (% of Frames Sampled)

Figure 5E displays the proportion of frames when the gaze was directed along the horizontal meridian for each condition in the 10 participants for whom eye tracking was recorded. ANOVA revealed a Gaze × Orientation interaction: *F*_(3,27)_ = 4.02, *p* = 0.017. Both factors also showed a main effect (both *p* < 0.007). No other interactions were found (all *p* > 0.051). The *post hoc* analyses focused on delineating the significant interaction. The analyses revealed that the interaction was driven by less gaze time along the horizontal meridian for the standard relative to the other orientations during the free gaze (all *p* < 0.001) condition. No orientation effects were found in the central fixation condition (*p* = 0.569). A main effect of Size was also found: *F*_(1,9)_ = 17.43, *p* = 0.002, denoting less gaze time along the horizontal meridian when the stimulus was large. In summary, the participants spent less gaze time along the horizontal meridian when the stimulus was oriented in its standard orientation and when the stimulus was large.

### Gaze Along the Vertical Meridian (% of Frames Sampled)

Figure 5F displays the proportion of frames when the gaze was directed along the vertical meridian for each condition in the 10 participants for whom eye tracking was recorded. ANOVA revealed Gaze × Size (*F*_(1,9)_ = 11.00, *p* = 0.009) and Size × Orientation (*F*_(3,27)_ = 5.49, *p* = 0.004) interactions. Main effects for each factor were also significant (*p* < 0.002). No other interactions were found (all *p* > 0.150). The *post hoc* analyses focused on delineating the significant interactions, which revealed the following effects: participants spent more time gazing along the vertical meridian during central fixation compared to the free gaze condition when the stimulus was small (*p* < 0.001) and large (*p* = 0.033) with the differences being more pronounced for the former, and when the stimulus was small compared to large in both the free gaze (*p* < 0.001) and central fixation (*p* < 0.001) conditions, with the differences being more pronounced for the latter. Participants also spent more time gazing along the vertical meridian in the standard and 180° orientations compared to the 90° and 270° orientations (all *p* < 0.027) and the 270° compared to the 90° orientation (*p* = 0.004) in the free gaze condition. Conversely, participants spent less time gazing along the vertical meridian in the 90° compared to 180° orientation in the central fixation condition (*p* = 0.048). We also carried out a paired *t*-test to assess differences between the proportion of frames where the gaze was directed along the vertical vs. horizontal meridians, which revealed that participants spent more time directing their gaze along the vertical compared to the horizontal meridians (*t*_(9)_ = 2.90, *p* = 0.017). Note that the means between the meridians do not add up to zero (Figures 5E,F) for the same reason described in the visual field experiment. Namely, participants spent a proportion of their time fixating at the center which encompasses both meridians. In summary, the participants spent more gaze time along the vertical meridian when the stimulus was either in its standard or 180° orientation in the free gaze condition and spent more gaze time overall along the vertical meridian in the central fixation compared to free gaze condition.

### Correlations between Illusion Susceptibility and Eye Tracking Measurements

We performed a series of correlations to determine if the susceptibility index for a given condition was associated with any of the eye-tracking measurements. The analyses revealed that the illusion increased in strength when participants made fewer saccades (*r*_(16)_ = −0.54, *p* = 0.031) and spent more time gazing along the vertical meridian (*r*_(16)_ = 0.71, *p* = 0.001). No associations were observed between the strength of the illusion and the other eye tracking measurements (gaze deviation from center: *r*_(16)_ = −0.37, *p* = 0.164; trial duration *r*_(16)_ = −0.19, *p* = 0.491; gaze along the horizontal meridian: *r*_(16)_ = 0.06, *p* = 0.826).

## Discussion

We characterized the effects of orientation, size and eye gaze on the strength of the vertical-horizontal illusion. As far as we know, this is the first eye tracking investigation of the illusion. Specifically, we were interested in determining whether or not the eye tracking might support Künnapas (1955) framing theory. We reasoned that a framing effect would not be possible without position constancy enabling a stable perceptual representation of the outside world. Thus, we assumed that the mechanisms of position constancy would be more taxed in conditions when the eyes moved more. Under these conditions, we hypothesized that susceptibility to the illusion would decrease. The results tend to support these predictions. In the ensuing discussion, we discuss methodological issues, summarize the findings, and explain how our experiments further our understanding of the vertical-horizontal illusion.

### Methodological Issues

Ideally, all 20 participants who performed the visual-field and vertical-horizontal illusion experiments should have had eye tracking. Instead, only half the participants did. The question then arises as to whether or not the group of participants who had eye tracking were representative of the others who did not have eye tracking. We would argue that they were except for one caveat. The caveat is that the experiments with eye tracking occurred between semesters in participants who received monetary compensation while those without eye tracking occurred during semester in participants who received course credit. It is possible that the participants who had eye tracking took the experiment more seriously, as evidenced by their higher accuracy scores in the visual-field experiment. We do not think that this matters too much for a number of reasons.

First, the only differences in procedures between participant groups was whether or not the experiments began with a calibration procedure and whether or not the eye tracker was turned on. Otherwise the procedures were identical. Both groups received the same instructions, performed the same task, used the same chin rest and viewed the same monitor at the same distance from the eyes. Second, independent *t*-tests performed on the illusion susceptibility measures in the vertical-horizontal illusion experiment did not reveal any group differences. Thus, any inference made about illusion susceptibility in one group also applies to the other. Third, it seems unlikely that two different types of eye movement patterns manifested by two different groups of participants could yield identical illusion susceptibility scores in the vertical-horizontal illusion. Thus, the eye-tracking data in the vertical-horizontal illusion experiment is most likely representative for both groups of participants.

Another issue is that the boundaries of the computer monitor, which has a similar aspect ratio as the visual field, could have contributed to the framing effect. This is certainly a possibility. Earlier studies have demonstrated that the illusion is stronger when an elongated artificial frame is presented over the figure in the horizontal compared to the vertical orientation (Künnapas, 1957; Prinzmetal and Gettleman, 1993). However, it should be underscored that the illusion is still quite robust without any artificial frame, as demonstrated when the stimulus is presented as a light in complete darkness (Prinzmetal and Gettleman, 1993). Furthermore, the existence of artificial frames, such as those produced by the boundaries of a computer monitor, has only emerged in recent history and would have not contributed to the evolutionary pressures causing us to see the vertical-horizontal illusion.

### Visual-Field Experiment: Confirming the Presence of a Framing Effect

The visual-field experiment aimed to determine how the shape of the visual field under binocular conditions might influence perceptual processing along the vertical and horizontal meridians and how attention may differ along these two meridians. This was achieved by comparing people’s ability to detect brief presentations of a target along the vertical vs. horizontal meridians at two different eccentricities. As one would predict from a framing effect, the participants seemed to have more difficulty detecting targets when they appeared along the vertical meridian in the periphery. Namely, accuracy was reduced when the target appeared in the lower visual field and periphery (Figure 4A) and reaction times were slower when the target appeared in the vertical meridian and periphery (Figure 4B). These results cannot be explained by speed-accuracy trade-off effects. When accuracy and reaction times for each participant were averaged across all conditions and correlated with each other, we did not find any evidence that accuracy decreased in the participants who responded more quickly. Unlike the vertical-horizontal illusion experiment, participants were required to always maintain fixation at the center of the screen during the visual-field experiment. The eye tracking results demonstrated that the participants were generally good at doing this—although more saccades were detected when the target appeared in the periphery (Figure 4D). It is important to note that these saccades did not affect the overall amount of gaze deviation from fixation (Figure 4C) and that the average number of saccade counts across conditions was on par with what is expected for frequencies of micro-saccade during periods of fixation (Pastukhov and Braun, 2010). The higher number of saccades in the periphery condition could reflect an increase in involuntary movements to move the eyes when a target appeared there. As predicted, the eye tracking data also revealed that participants spent more time gazing along the vertical compared to the horizontal meridian (Figures 4E,F). We speculate this may relate to the framing effect demonstrated by the accuracy and reaction time measures described earlier. Alternatively, this could relate to the possibility that the brain treated the vertical line as a perspective cue. Indeed, perspective cues in the real world draw people’s attention more often along the vertical meridian than they do along the horizontal meridian (Gregory, 1968, 1998). We will expand on this idea later when we discuss the vertical-horizontal illusion experiment.

The finding that participants were faster at detecting the target along the horizontal compared to the vertical meridian confirms the presence of a framing effect. However, a framing effect would also predict better performance for detecting a target in the lower compared to the upper visual field, given that the extent of the upper is smaller than the lower visual field (Niederhauser and Mojon, 2002). Our results demonstrated the reverse pattern of results. Namely, accuracy was reduced for the lower visual field.

To understand this result, one should consider both the demands of the task and the functional organization of the visual system. In terms of task demands, the participant had to differentiate a target that differed physically in color as well as luminance from three distracter stimuli. In terms of the functional organization of the visual system, the dorsal stream receives stronger retinal inputs from the lower visual field while the ventral stream receives stronger retinal inputs from the upper visual field (Engel et al., 1997; Wandell and Winawer, 2011; Chouinard et al., 2012). The dorsal stream also receives stronger retinal inputs from the magnocellular system, which exhibits high-contrast sensitivity and conveys achromatic information with low spatial frequencies, than it does from the parvocellular system, which exhibits low-contrast sensitivity and conveys chromatic information with high spatial frequencies (Schiller et al., 1990; Zemon and Gordon, 2006; Nassi and Callaway, 2009). Taken together, we speculate that our finding relates to differences in the sensitivity to detect color and luminance differences between the lower and upper visual fields, with the lower visual field being less sensitive.

### Vertical-Horizontal Illusion Experiment: New Insights

The vertical-horizontal illusion experiment aimed to determine the effects of orientation, size and eye gaze on the strength of the illusion. One of the two lines of the stimulus was designated as the standard feature while the other was designated as the comparison feature. The stimulus varied in size (small vs. large) and orientation (standard, 90°, 180°, or 270°) while the participant adjusted the comparison feature to match the standard feature. This was done while the participant either maintained fixation where the two lines bisected or free gazed wherever they liked on the computer screen.

Based on the framing theory, we hypothesized that the illusion would be stronger when the stimulus is presented in either its standard inverted T orientation or when it is rotated 180° compared to other orientations. As it turns out, this is precisely what we found (Figure 5A), which replicates a consistent result reported in a number of studies (Finger and Spelt, 1947; Künnapas, 1955; Avery and Day, 1969; Deregowski and Ellis, 1972; Harris et al., 1974; Thompson and Schiffman, 1974; Harris et al., 1974). In addition, we demonstrate that the illusion was stronger when it was small (Figure 5A), which also replicates previous research (Thompson and Schiffman, 1974). Orientation no longer had an effect when the stimulus was small, presumably because the framing effect diminished as the stimulus got smaller.

The effects of cortical magnification could potentially explain some of the differences between the small and large conditions during the central fixation condition, given that the ends of the two lines were located at higher eccentricities for the latter relative to the former. Any differences in perceived length between the two lines might have been magnified as stimuli got smaller and more cortical resources became devoted to processing the ends of the two lines.

Could differences in cortical magnification between the vertical and horizontal meridians explain the vertical-horizontal illusion? We think this is a possibility that requires further investigation. The mapping of the retina to early areas of the visual cortex (V1 and V2) with functional magnetic resonance imaging (fMRI) demonstrates that cortical magnification is different for the horizontal meridian, which is represented within the boundaries of V1, and the vertical meridian, which is represented along the boundaries between V1 and V2. At a given eccentricity, more cortical surface area is devoted for processing information along the vertical compared to the horizontal meridians (Liu et al., 2006). There is also a spatial shift in cortical representation whereby the vertical meridian is represented by more anterior cortical tissue (Liu et al., 2006). Why would the meridians be represented this way in the early visual cortex? The answer has to do with the amount of cortical space available for representing different ranges of eccentricities. If there is a limited amount of cortical tissue (X) and there is a wider range of eccentricities to represent along the horizontal (A) than the vertical (B) meridians then there is going to be more cortical tissue available for a particular eccentricity for the latter. In mathematical terms: X/A is smaller than X/B because A > B. How these differences in cortical representations might impact size perception is unclear, although some fMRI studies demonstrate how the apparent image size of a stimulus can invoke more anterior activation in the visual cortex as it becomes larger even when the retinal image size is held constant (Murray et al., 2006; Sperandio et al., 2012; Sperandio and Chouinard, 2015).

For the first time, we demonstrate how the illusion is stronger when participants were asked to maintain fixation compared to when they were permitted to free gaze. As shown in Figure 5A, the illusion was strongest when the participants were asked to maintain fixation in the small condition. Furthermore, the correlation analyses revealed that the illusion was stronger in conditions when participants made fewer saccades. Thus, as hypothesized, greater retinal image stability translates to increases in susceptibility to the illusion, which we think may relate to the ease with which position constancy is computed and the importance of these mechanisms for establishing a frame denoting the boundaries of a perceptual visual field even when small eye movements are made. It is also conceivable that when eye movements are large enough, such as in the free gaze condition, the perceived location of these boundaries would cease to be stable, diminishing further framing effects on the vertical-horizontal illusion.

The results revealed other interesting and informative effects of eye gaze on illusion strength that should be considered. First, free gazing caused participants to gaze more along the vertical than the horizontal meridian when the stimulus appeared in the standard and 180° orientations compared to the 90° and 270° orientations (Figure 5F). Second, the amount of gaze deviation from the center of the computer screen for the large stimulus during free gazing was highest when the stimulus appeared in its standard orientation (Figure 5B). Taken together, these findings suggest that the bisector line draws people’s attention along the vertical meridian, particularly when the stimulus is presented in its standard orientation. The question then arises as to why this is the case and why more gazing along the vertical meridian would translate to an increase in the strength of the illusion. One possibility is that the bisector line could be treated as a perspective cue, causing the brain to perceptually rescale its length.

Others have shown that the strength of the vertical-horizontal illusion can be modulated as a function of how realistic the bisector line simulates perspective cues in the real world (Girgus and Coren, 1975; Wolfe et al., 2005). The possibility that the bisector line might be treated as a perspective cue was first proposed by Gregory (1968, 1998). According to this theory, the illusion is based on the misapplication of constancy scaling mechanisms that would normally achieve size constancy in the real world. Namely, perspective cues in the real world often draw our attention towards the upper visual field. Likewise, the bisector line in the illusion causes the brain to mistake it as a perspective cue, signaling depth towards the upper visual field. As a consequence, the brain perceptually rescales this line as being longer than its retinal image size. Although the misapplied constancy scaling theory may help explain why attention was directed along the vertical meridian most strongly in the free gaze condition when the illusion was large and oriented in its standard configuration (Figure 5B), the theory does not explain why the illusion was as strong when it was rotated 180°. On the other hand, according to the framing theory, the strength of the illusion should be similar for both orientations.

### Avenues for Future Research

Our study was not designed to evaluate different theories. Rather, it aimed to determine the effects of retinal image stability on the vertical-horizontal illusion. We thought that this would be informative given that the best known theory for explaining the illusion, the framing theory, arguably predicts greater susceptibility as retinal image stability increases. It is conceivable that the perceptual representation of the shape of the visual field, which creates a frame for the effect to occur, is not as salient as more eye movements are made. Future work could consider the results from this investigation in the design of future experiments to test more specific questions. Such questions could pit different theories against each other, to explore further how spatial attention is allocated between the bisector and bisected lines, and explore further how differences in spatial attention might correlate with illusion strength.

## Author Contributions

HJP contributed to the data collection and the writing of the manuscript. PAC and OL contributed in the design, data collection, data analysis and the writing of the manuscript.

## Funding

This work was supported by La Trobe University’s Understanding Disease and Building Healthy Communities Research Focus Areas grants awarded to PAC.

## Conflict of Interest Statement

The authors declare that the research was conducted in the absence of any commercial or financial relationships that could be construed as a potential conflict of interest. The reviewer FF and handling Editor declared their shared affiliation, and the handling Editor states that the process nevertheless met the standards of a fair and objective review.
